# YTHDF1 Promotes Cyclin B1 Translation through m^6^A Modulation and Contributes to the Poor Prognosis of Lung Adenocarcinoma with KRAS/TP53 Co-Mutation

**DOI:** 10.3390/cells10071669

**Published:** 2021-07-02

**Authors:** Xiaoying Lou, Jinfeng Ning, Wei Liu, Kexin Li, Benheng Qian, Danfei Xu, Yue Wu, Donghong Zhang, Wei Cui

**Affiliations:** 1 National Cancer Center/National Clinical Research Center for Cancer/Cancer Hospital, State Key Laboratory of Molecular Oncology, Department of Clinical Laboratory, Chinese Academy of Medical Sciences and Peking Union Medical College, Beijing 100021, China; lxy15723077967@163.com (X.L.); likexin0813@student.pumc.edu.cn (K.L.); xudanfei_pumch@hotmail.com (D.X.); wwuyuey@163.com (Y.W.); 2 The Thoracic Department of Harbin Medical University Cancer Hospital, 150 Haping Road, Harbin 150040, China; ningjinfenghyd@icloud.com; 3 The Fourth Department of Medical Oncology, Harbin Medical University Cancer Hospital, 150 Haping Road, Harbin 150040, China; 13945147542@163.com; 4 Department of Cardiology, The Second Affiliated Hospital of Wenzhou Medical University, 109 Xueyuan Road, Wenzhou 325027, China; 18516196271@163.com; 5 Center for Molecular and Translational Medicine, Research Science Center, Georgia State University, Atlanta, GA 30303, USA

**Keywords:** lung adenocarcinoma, KRAS/TP53 mutation, YTHDF1, cyclin B1, m^6^A modification

## Abstract

KRAS and TP53 mutations are the two most common driver mutations in patients with lung adenocarcinoma (LUAD), and they appear to reduce latency and increase metastatic proclivity when a KRAS and TP53 co-mutation (KRAS/TP53-mut) occurs. However, the molecular mechanism involved is unclear. *N*^6^-methyladenosine (m^6^A), the most abundant RNA modification in mammal mRNAs, plays a critical role in tumorigenesis. Here, we used genomic and transcriptomic data and found that only LUAD patients with KRAS/TP53-mut, but not an individual mutation, appeared to exhibit poor overall survival when compared with patients without KRAS and TP53 mutation (wildtype). Subsequently, we analyzed the differential expression of the 15-m^6^A-related genes in LUAD with different mutations and found that YTHDF1 was the most upregulated in KRAS/TP53-mut patients and associated with their adverse prognosis. Bioinformatics and experimental evidence indicated that elevated YTHDF1 functionally promoted the translation of cyclin B1 mRNA in an m^6^A-dependent manner, thereby facilitating the tumor proliferation and poor prognosis of LUAD with KRAS/TP53-mut. Furthermore, the concurrent increase in YTHDF1 and cyclin B1 was confirmed by immunohistochemistry staining in patients with co-occurring KRAS/TP53 mutations. YTHDF1 was correlated with an unfavorable clinical stage and tumor size. Collectively, we identified and confirmed a novel “YTHDF1–m^6^A–cyclin B1 translation” axis as an essential molecular pathway for the prognosis of KRAS/TP53-mut LUAD.

## 1. Introduction

Over the past 15 years, subgroups of LUAD have been identified by using oncogenic drivers, such as the gene encoding epidermal growth factor receptor (EGFR), resulting in breakthroughs for the treatment of non-small-cell lung cancer (NSCLC) [1]. However, patients driven by the oncogenic alteration of KRAS can vary considerably in their histological appearance and immunohistochemical profile [2]. Meanwhile, mutations in tumor suppressor genes TP53 and STK11 are common in LUAD and frequently co-occur with the KRAS mutation [3,4,5]. Recent studies have demonstrated that the co-occurrence of TP53 and STK11 has a profound influence on the molecular and clinical heterogeneity of KRAS-driven lung cancers. An oncogenic KRAS-mutant with p53 loss can induce LUAD with reduced latency and increased metastatic proclivity [6]. This raises the question of whether co-mutations impact the prognosis of LUAD.

As the most prevalent type of modification of an internal mRNA/lncRNA observed in a wide range of organisms, m^6^A has emerged as a potential new mechanism of post-transcriptional gene regulation [7,8,9,10]. The biological functions of m^6^A are mediated by m^6^A methyltransferases (“writers”), m^6^A demethylases (“erasers”), and m^6^A-specific binding proteins (“readers”) [11]. Since m^6^A modification has been reported to play a critical role in RNA translation, stability, and alternative splicing, the abnormal expression of m^6^A components is associated with cancer development and progression [12,13,14]. Our previous study reported that elevated leukocyte m^6^A in LUAD patients could significantly discriminate them from healthy individuals [15]. In addition, our recent study found that the suppression of m^6^A modification by the demethylase of ALKBH5 aggravates the oncological behavior of LUAD with co-mutant KRAS and LKB1. LKB1 loss upregulates ALKBH5 via DNA hypermethylation and, subsequently reduces m^6^A modification of some critical oncogenes, such as MYC, SOX2, and SMAD7, thus further increasing their stability via the YTHDF2 pathway [16]. YTHDF1, as an m^6^A reader protein, could directly promote the translation of methylated mRNAs. Elevated YTHDF1 has been found in most cancer cells [10,14,17,18,19]. Deficiency of YTHDF1 functionally prevents NSCLC cell proliferation and tumor formation through inhibiting the translational efficiency of cyclin-dependent kinase 2 (CDK2), cyclin-dependent kinase 4 (CDK4), and cyclin D1 (CCND1). Furthermore, YTHDF1 could regulate lung cancer cells responding to cisplatin-dependent chemotherapy as well as affect the patients’ treatment and prognosis [14]. Therefore, we hypothesized that YTHDF1 may play a vital role in LUAD along with co-mutant KRAS/TP53 through an m^6^A-dependent pathway.

CCNB1, as a well-known cell-cycle regulator, has been repeatedly reported in most cancers, including LUAD [20,21]. Upregulated CCNB1 in NSCLC closely correlates with tumor growth, differentiation, and vascular invasion [22,23]. Silencing of CCNB1 exerts an antitumor effect on melanoma lung metastasis [24]. Administration of an inhibitor of histone deacetylases (HDCAi) and silibinin could promote proteasomal degradation of CCNB1, resulting in G2/M arrest and apoptotic cell death [25]. In addition, high expression of CCNB1 in NSCLC leads to short overall survival [22,23,26].

In this study, we firstly observed that increased YTHDF1 expression was associated with the poor prognosis of LUAD patients with KRAS and TP53 co-mutation. A multi-omics analysis using TCGA and TCPA databases demonstrated that mRNA expression of YTHDF1 was associated with the translation efficiency of CCNB1 in co-mutant KRAS/TP53 lung cancer. Subsequently, we further validated that YTHDF1 promoted the proliferation of co-mutant KRAS/TP53 lung cancer cells by promoting the translation of CCNB1 in vitro. Furthermore, YTHDF1 was confirmed to be correlated with CCNB1 in patients with KRAS and TP53 co-mutation. Together, our data highlighted the critical role played by the m^6^A reader, YTHDF1, and identified a promising marker of therapeutic significance in co-mutant KRAS/TP53 lung cancer.

## 2. Materials and Methods

### 2.1. Online Database

The transcriptome, somatic mutation, and clinical data were downloaded from UCSC (http://xena.ucsc.edu/, 14 December 2020). GSE72094 was downloaded from GEO dataset. The GSE72094 cohort recruited 442 patients with detailed mRNA expression data and EGFR/KRAS/TP53/STK11 Sanger sequencing analysis [27]. LUAD samples were divided into 9 groups with more than 10 samples in each on the basis of the TP53, KRAS, EGFR, and STK11 mutation status. The number of mutation subgroups is shown in Tables S1 and S2 (Supplementary Materials). The wildtype group was defined as patients without either of the four mutations. Data on the proteins were downloaded from TCPA database, and missing values were imputed using the nearest-neighbor averaging method of the impute R package (Supplementary Materials Table S3). The protein name was transformed into gene name for RNA expression extraction (Supplementary Materials Tables S4 and S5). A total of 150 non-phosphorylated proteins were subsequently analyzed to determine the differences in both the translation efficiency and the protein level between the KRAS/TP53-mut and wildtype groups (Supplementary Materials Tables S6 and S7). The translation efficiency was calculated using the following formula: translation efficiency = protein_i_/RNA_i_ (i refers to each non-phosphorylated protein).

### 2.2. Human Samples

LUAD tissues were acquired from 70 lung cancer patients who underwent curative surgical resection at the Harbin Medical University Cancer Hospital (Harbin Medical University, Harbin, China). This study was approved by the Ethics Committees of the National Cancer Center/Cancer Hospital, Chinese Academy of Medical Sciences, and Peking Union Medical College (Beijing, China). All patients provided written informed consent before enrollment.

### 2.3. Gene Set Enrichment Analysis (GSEA) 

The JavaGSEA software was used to determine the upregulated or downregulated pathways in the KRAS/TP53-mut group, compared with the wildtype group [28]. A gene set at a nominal *p*-value of ≤0.05 was considered to indicate a significantly enriched pathway. The upregulated pathways in KRAS/TP53-mut and wildtype groups are presented in Tables S8 and S9 (Supplementary Materials).

### 2.4. Cell Culture and siRNA Transfection

H1650, H1395, A427, A549, H1299, Calu3, H2009, and H1792 cells were purchased from the American Type Culture Collection (ATCC) in USA. The cells were cultured in a medium containing 10% fetal bovine serum, 1% penicillin, and 1% streptomycin at 37 °C in a 5% CO_2_ atmosphere. Scramble siRNA (Si-CN, sc-37007) and siRNA-YTHDF1 (Si-YTHDF1, sc-76945, sc-76945-PR) were ordered from Santa Cruz (Pierce, USA). The cells were transfected with the siRNA using Lipofectamine RNAiMAX Transfection Reagent (cat: 13-778-075, Invitrogen) following the manufacturer’s instructions.

### 2.5. RNA Isolation and RT-qPCR

Total RNA was extracted from lung cancer cells using TRIzol reagent (Life Technologies, Gaithersburg, MD, USA), following the manufacturer’s instructions. For RT-qPCR, the RNA was reverse-transcribed into cDNA using a reverse transcription kit (Takara, Otsu, Shiga, Japan). PCR amplification reactions were performed in duplicate using SYBR Green PCR Master Mix (Applied Biosystems). Quantification was conducted using the ΔΔCT method, and data were normalized to the expression level of GAPDH. The primers used in the RT-qPCR were as follows: YTHDF1 forward, 5′–ATACCTCACCACCTACGGACA–3′ and reverse, 5′–GTGCTGATAGATGTTGTTCCCC–3′; CCNB1 forward, 5′–AATAAGGCGAAGATCAACATGGC–3′ and reverse, 5′–TTTGTTACCAATGTCCCCAAGAG–3′; GAPDH forward, 5′–ACAACTTTGGTATCGTGGAAGG–3′ and reverse, 5′–GCCATCACGCCACAGTTTC–3′.

### 2.6. Immunoblotting

The treated cells were homogenized on an ice-cold RIPA Lysis (Thermo Fisher Scientific, Waltham, MA, USA) and quantified using a BCA assay kit (Pierce, Santa Cruz, CA, USA). The proteins were fractionated using SDS-PAGE, transferred onto polyvinylidene fluoride (PVDF) membranes, blocked with 5% nonfat milk in TBST, and then blotted with specific antibodies at room temperature overnight. The antibodies used were as follows: anti-YTHDF1 (1:400, Proteintech 17479-1-AP), anti-CCNB1 (1:500, Santa Cruz Biotechnology sc-245), and anti-GAPDH (1:2000, Santa Cruz Biotechnology sc-47724). The bands were visualized using an enhanced chemiluminescence detection kit (Thermo Fisher Scientific, Waltham, MA, USA).

### 2.7. Immunohistochemical (IHC) Staining and Analysis

Following the methods detailed in our previous reports [29,30], human tissue samples were obtained and fixed in 10% formalin, dehydrated, and embedded in paraffin. Then, 4 mm thick tissue slices were cut, dried, deparaffinized, and treated in a boiling citrate buffer (pH 6.0) for a total of 15 min. The slides were blocked with a peroxidase block (DAKO, Carpinteria, CA) and 2.5% horse serum for 1 h. Then, the slides were incubated overnight with primary antibodies against YTHDF1 and CCNB1 (dilution 1:500) at 4 °C. Thereafter, the slides were stained using an EnVision+ Dual Link System HRP (Dako K406311-2), counterstained with hematoxylin, and mounted. The histoscore (H-score) was calculated as the product of the intensity of staining (graded as follows: 0, no staining; 1, weak; 2, median; 3, strong) and percentage of positive cells. The range of possible scores was from 0 to 300.

### 2.8. RNA Immunoprecipitation Assay and RT-qPCR Analysis

The total RNA was isolated using the TRIzol method. Polyadenylated RNA isolated from the cells indicated was fragmented into ~100 nt sections using the NEBNext Magnesium RNA Fragmentation Module (NEB E6150S). RNA was incubated with the m^6^A, YTHDF1, or IgG antibody (5 μg) for immunoprecipitation overnight at 4 °C, according to the standard protocol of the EpiMark *N*^6^-Methyladenosine Enrichment Kit (NEB E1610S). The enrichment of certain fragments was determined using real-time PCR. The enrichment of the RNAs was normalized to inputs from the siRNA control group [31,32].

### 2.9. Protein Stability

To evaluate protein stability, H1792 cells were treated with 100 ug/mL cycloheximide (CHX, Sigma 01810) at indicated times and harvested. The protein expression level of CCNB1 was then determined through Western blot analysis.

### 2.10. Cell Proliferation Assays

Cell proliferation was measured by conducting a Cell Counting Kit-8 (CCK-8, Sigma 96992) assay and colony formation assay. For the CCK8 assay, siRNA-transfected cells were cultured in a 96-well plate. Cell viability was determined every 24 h. The plate was incubated at 37 °C for 2 h, and 10 μL of CCK-8 solution was added to each well. Then, spectrophotometric absorbance was measured at 450 nm in each sample. All experiments were performed in triplicate.

For the colony formation assay, a certain number of control or transfected cells were plated into a 6-well plate and maintained in culture medium containing 10% FBS for 2 weeks. The colonies were fixed using methanol and stained using crystal violet (Sigma C0775). The colony number was determined by counting the number of stained colonies using the ImageJ software.

### 2.11. Statistical Analysis

All statistical analyses were performed using the R software (version 3.6.2, www.r-project.org, 10 February 2020). Continuous variables were expressed as the mean ± standard deviation and were analyzed using Student’s *t*-test, Wilcoxon test, or Kruskal–Wallis test. Categorical variables were compared using the chi-square test or Fisher’s exact test. The correlation analysis was carried out using the Spearman method, which was performed with the “cor.test” function. Survival analysis was performed using the Kaplan–Meier method, and data were compared using log-rank tests with the “survival” and “survminer” packages. A *p*-value < 0.05 was considered to indicate a statistically significant difference in all tests.

## 3. Results

### 3.1. Increased YTHDF1 Expression Is Associated with a Poor Prognosis in LUAD Patients with KRAS and TP53 Co-Mutation

**Figure 1:**
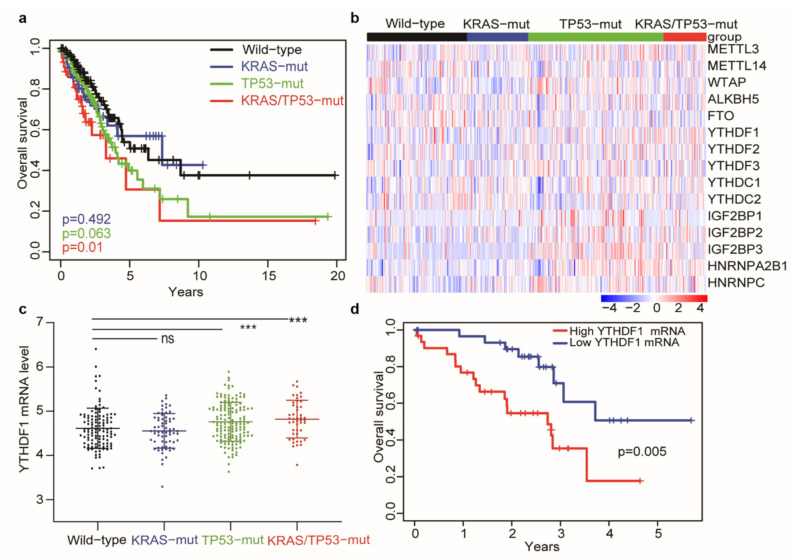
YTHDF1 is upregulated in KRAS/TP53-mutant lung cancer and is indicative of a poor prognosis. (**a**) Kaplan–Meier survival plots for KRAS/TP53-mut (*n* = 44), KRAS-mut (*n* = 64), and TP53-mut (*n* = 146) groups compared with the wildtype (*n* = 106) group of the LUAD cohort. (**b**) Heatmap of mRNA expression of the m^6^A-related genes in the wildtype (*n* = 108), KRAS-mut (*n* = 66), TP53-mut (*n* = 147), and KRAS/TP53-mut (*n* = 44) groups of the LUAD cohort. (**c**) Plot showing the mRNA expression of YTHDF1 in four different mutation groups. (**d**) Kaplan–Meier survival plots for male LUAD patients stratified by quantile (0.25 and 0.75). The *p*-values were determined for the Exh result using the log-rank test or Wilcoxon rank-sum test (*** *p* < 0.001).

The KRAS and TP53 co-mutation appears to reduce latency and increase metastatic proclivity [6]. To ascertain the impact of TP53 and KRAS mutation on the prognosis of LUAD patients, we performed a Kaplan–Meier survival analysis on four mutation groups (wildtype, KRAS-mut, TP53-mut, and KRAS/TP53-mut) using 367 LUAD samples from The Cancer Genome Atlas (TCGA) database. Compared with the wildtype group, patients with the KRAS and TP53 co-mutation exhibited the worst overall survival (*p* = 0.01, Figure 1a). However, no differences were observed between the KRAS-mutated or TP53-mutated group and the wildtype group (Figure 1a).

Increasing evidence has elucidated the critical role of m^6^A RNA modification in the progression of lung cancer [12,13,14,33,34]. We next investigated the correlation between TP53 and/or KRAS mutation and m^6^A-related genes in LUAD. The heatmap depicted the differential expression of 15-m^6^A-related genes in the four groups (Figure 1b and Supplementary Materials Figure S1). Interestingly, the mRNA expression of YTHDF1 was the most elevated in the KRAS/TP53-mut group (Figure 1c). Furthermore, we performed univariate and multivariate cox regression analysis including 288 lung adenocarcinomas with a KRAS and/or TP53 mutation. The results indicated gender (male) is an independent prognostic factor for 288 patients (data not shown). Therefore, we further performed a stratified survival analysis and identified that a high level of YTHDF1 exhibited an adverse clinical outcome of male LUAD with KRAS and TP53 co-mutation (Figure 1d, *p* = 0.005). Taken together, these results suggested that a high expression of YTHDF1 is associated with a poor prognosis of co-mutant KRAS/TP53 lung cancer.

### 3.2. YTHDF1 Expression Is Associated with the Translation of Cyclin B1 and Cell Proliferation in Co-Mutant KRAS/TP53 Lung Cancer

**Figure 2:**
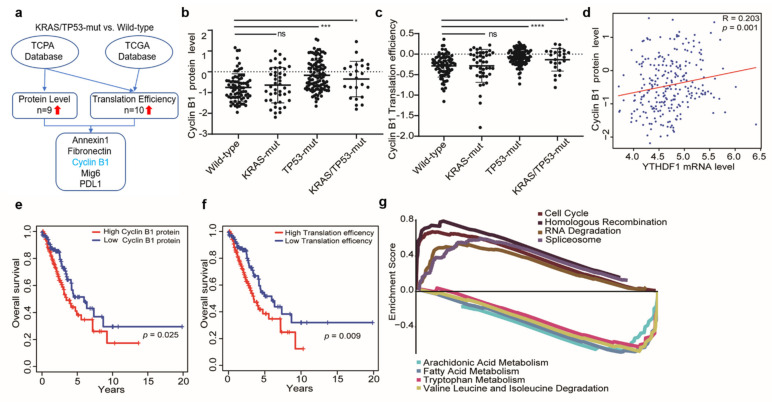
YTHDF1 is associated with the translation of CCNB1 and cell proliferation in co-mutant KRAS/TP53 lung cancer. (**a**) Flow chart for screening the differential translation efficiency between co-mut KRAS/TP53 and wildtype LUAD patients. (**b**,**c**) Plot showing the protein level (**b**) and translation efficiency (**c**) of cyclin B1 in four different mutation groups. (**d**) Spearman’s rank correlation between the YTHDF1 mRNA level and the cyclin B1 protein level in LUAD patients (*n* = 357). (**e**,**f**) Kaplan–Meier survival plot of LUAD patients grouped by the protein (**e**) and translation efficiency (**f**) patterns of cyclin B1 with a cutoff of median expression. (**g**) Multi-GSEA plot showing the top eight pathways enriched in KRAS/TP53-mut patients compared with the wildtype group. The *p*-values were calculated using the log-rank test or Wilcoxon rank-sum test (* *p* < 0.05; *** *p* < 0.001; **** *p* < 0.0001).

YTHDF1 has been proven to actively promote protein synthesis by interacting with translation machinery in an m^6^A-dependent manner [10]. Therefore, we compared the differential global gene expression at the protein and mRNA levels from TCPA and TCGA databases (Figure 2a). The translation efficiency was defined as the ratio of protein to mRNA expression, and translation efficiency of each gene was compared between the KRAS/TP53-mut group and the wildtype group. As shown in Figure 2a, we found a concurrent increase in protein level and translation efficiency of 5genes (Annexin1, Fibronectin, Cyclin B1, Mig6, PDL1) in the KRAS/TP53-mut group compared with the wildtype group (Figure 2b,c), which is related to carcinogenesis. Especially, the protein level of cyclin B1 was positively correlated with YTHDF1 mRNA expression (Figure 2d, coefficient = 0.203, *p* = 0.001). In addition, the survival analysis indicated that the high protein level and translation efficiency of cyclin B1 were correlated with poor overall survival of LUAD patients (*p* = 0.025 and *p* = 0.009; Figure 2e,f).

To identify major pathways involved in the poor prognosis of patients with KRAS/TP53-mut, we conducted a gene set enrichment analysis (GSEA) assay. The result demonstrated the top eight prominent enriched signatures, including the cell cycle, homologous recombination, RNA degradation, and spliceosome, which were significantly upregulated in the KRAS/TP53-mut group, whereas several metabolism-related pathways, including arachidonic acid metabolism, fatty acid metabolism, were downregulated (Figure 2g, Supplementary Materials Tables S8 and S9). Together, our results indicated that YTHDF1 is associated with the translation efficiency of cyclin B1, which promotes cell proliferation in co-mutant KRAS/TP53 lung cancer.

### 3.3. YTHDF1 Promotes the Translation of Cyclin B1 and Proliferation via an m^6^A-Dependent Pathway in Co-Mutant KRAS/TP53 Lung Cancer Cells

**Figure 3:**
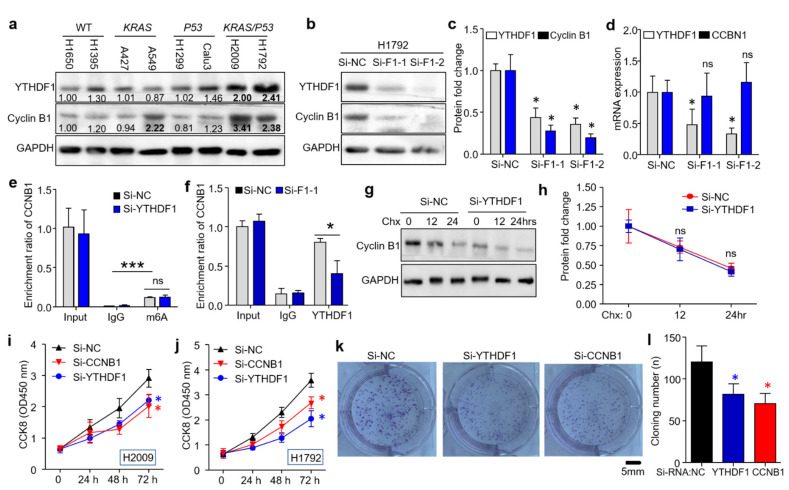
YTHDF1 regulates the translation of cyclin B1 in an m^6^A-dependent manner. (**a**) Western blotting analysis displaying the protein levels of YTHDF1 and cyclin B1 in the four types of cell lines with different mutations. (**b**,**c**) Western blotting showing the protein levels of cyclin B1 in H1792 cells transfected with an siRNA control or si-YTHDF1. (**d**) Relative mRNA level of CCNB1 in H1792 cells upon YTHDF1 knockdown. (**e**) m^6^A-RIP-qPCR validation of m^6^A abundance in the control and YTHDF1-knockdown cells. Input and IgG were used as the positive and negative controls, respectively. (**f**) YTHDF1-RIP-qPCR analysis of the enrichment of CCNB1 mRNA on YTHDF1 following knockdown of YTHDF1 in H1792 cells. (**g**,**h**) Western blotting analysis of cyclin B1 protein decay with CHX treatment in H1792 cells. (**i**,**j**) A CCK8 assay was conducted to determine the cell viability after YTHDF1 knockdown in H2009 and H1792 cells. (**k**,**l**) Representative images (**k**) and quantification (**l**) of the colony formation assay conducted on H1792 cells transfected with si-NC, si-YTHDF1, and si-CCNB1. Data are presented as the mean ± SD (*n* = 3/group). * *p* < 0.05; *** *p* < 0.001. The *p*-values were determined by conducting a Student’s *t*-test.

To further confirm the regulation mechanism of YTHDF1 on cyclin B1 translation, we first screened the expression profile of YTHDF1 and cyclin B1 across the common lung cancer cell lines with different mutation states (wildtype, KRAS-mut, TP53-mut, and KRAS/TP53-mut). Western blotting showed that the protein levels of YTHDF1 and cyclin B1 were significantly elevated in the KRAS/TP53-mut cell lines (H2009 and H1792 cells, Figure 3a). Then, the H1792 cells were transfected with two independent siRNAs (Si-YTHDF1-1 and Si-YTHDF1-2) to selectively knock down YTHDF1. Interestingly, the silencing of YTHDF1 remarkably decreased the cyclin B1 protein but not the mRNA level (Figure 3b–d). To test if YTHDF1 regulates cyclin B1 via an m^6^A-dependent pathway, we performed m^6^A-RIP and assessed the effect of m^6^A modification on cyclin B1 mRNA. As expected, m^6^A could occupy the 3′UTR region of CCNB1 mRNA, regardless of YTHDF1 silencing (Figure 3e). Similarly, YTHDF1 could also immunoprecipitate on the m^6^A-modified region of CCNB1 mRNA, and this precipitation was reduced by YTHDF1 knockdown in H1792 cells (Figure 3f). As the protein level but not the mRNA level of CCNB1 decreased as a result of silencing YTHDF1, we assumed that YTHDF1 might regulate either the protein stability or the translation efficiency of cyclin B1. Then, we silenced YTHDF1 in H1792 and treated the sample with the protein translation inhibitor cycloheximide (CHX). Western blotting analysis revealed that the decay rate of cyclin B1 protein did not change upon silencing of YTHDF1. These observations confirmed that YTHDF1 affects the translation of cyclin B1 via an m^6^A modification mechanism (Figure 3g,h). To define the functional roles of YTHDF1 in cell proliferation, CCK8 and colony formation assays were conducted in H2009 and H1792, respectively. Consistent with CCNB1, depletion of YTHDF1 significantly decreased the viability of the H2009 and H1792 cells, as reflected by the increase in the expedited growth rate (Figure 3i,j). Similarly, YTHDF1 deletion also reduced the colony numbers of H1792 cells (Figure 3k,l). Overall, these results demonstrated that knockdown of YTHDF1 potentially suppressed the proliferation of KRAS/TP53-mut lung cancer via inhibiting the translation of cyclin B1.

### 3.4. The Correlation of YTHDF1 with Cyclin B1 Was Confirmed in Clinical LUAD Patients with Co-Mutant KRAS/TP53

**Figure 4:**
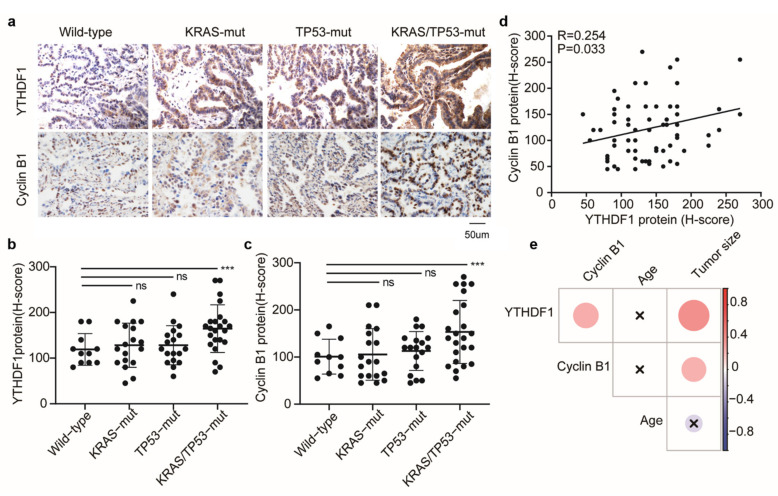
High expression of YTHDF1 in the KRAS/TP53-mut group was correlated with cyclin B1, unfavorable clinical stage, and tumor size. (**a**) Representative images of IHC staining for YTHDF1 and cyclin B1 expressions in tumor sections from human lung cancer specimens of the four different mutation groups. (**b**,**c**) Comparison of the IHC staining score of YTHDF1 (**b**) and cyclin B1 (**c**) in wildtype (*n* = 11), KRAS-mut (*n* = 18), TP53-mut (*n* = 18), and KRAS/TP53-mut (*n* = 23) groups. (**d**) Correlation analysis of the YTHDF1 H-score and cyclin B1 H-score (*n* = 70). (**e**) Correlation heatmap of the clinical characteristics in 70 cases of LUAD patients. *p* < 0.01; *** *p* < 0.001.

To confirm the association between YTHDF1 and cyclin B1 expression, 70 LUAD tissues were collected from patients with KRAS and/or TP53 mutation (Supplementary Materials Table S10). The IHC staining results demonstrated that the H-scores of both YTHDF1 and cyclin B1 were significantly elevated *(p* < 0.001) in the KRAS/TP53-mut group compared with the wildtype group (Figure 4a–c). Interestingly, there was a positive correlation between YTHDF1 and cyclin B1 (*R* = 0.254, *p* = 0.033; Figure 4d).

Then, we investigated the potential clinical relevance of YTHDF1 expression in LUAD patients. The correlation heatmap showed that YTHDF1 H-score was positively associated with cyclin B1 H-score (*R* = 0.25, *p* = 0.03) and tumor size (*R* = 0.38, *p* = 0.001). Meanwhile, the cyclin B1 H-score was positively associated with tumor size (*R* = 0.24, *p* = 0.046). However, neither the H-score of YTHDF1 nor that of cyclin B1 was associated with the age of LUAD (Figure 4e). In addition, stratification analysis showed that upregulation of YTHDF1 was associated with an unfavorable pathological stage but had no correlation with differentiation grade, TNM stage, or lymph node metastasis (Supplementary Materials Table S10). Taken together, the correlation between YTHDF1 and cyclin B1 expression was confirmed in patients with co-mutant KRAS/TP53, and upregulation of YTHDF1 was found to be associated with an unfavorable pathological stage and tumor size.

## 4. Discussion

Despite being one of the first oncogenic drivers discovered, targeted therapies for KRAS are still not adequately effective. The heterogeneity of KRAS-mutant NSCLC is partially a result of additional genetic alterations. Recent studies have divided KRAS-mutant NSCLC into different clusters, and the distinguishing feature of one of these clusters is the co-mutation of STK11 and TP53 [35]. Our survival analysis showed that a co-mutation of KRAS and TP53 appeared to have a worse prognosis than the wildtype in the LUAD cohort. Meanwhile, no differences were observed between the KRAS or TP53 mutation groups and the wildtype group. These results indicated that the presence of major co-mutations is of prognostic significance and predictive value for therapeutic vulnerabilities [36].

In eukaryotic cells, the control of mRNA translation and degradation is critical for the management of the quantity and duration of gene expression. m^6^A modification is important for regulating RNA translation, and it has been proven to participate in the regulation of cell growth, differentiation, and self-renewal [34,37,38]. Recent studies have reported that m^6^A participated in the initiation and progression of several cancers [39,40,41]. In particular, m^6^A-binding proteins, including m^6^A reader YTHDF1, modulate translation and the stability of the targeted RNAs. In various cancer cells, individual m6A “readers” affect gene expression by binding to m^6^A modification sites of specific mRNAs [42,43]. Here, we revealed that YTHDF1 was significantly upregulated in the KRAS/TP53-mut group, compared with the wildtype group. The upregulation of YTHDF1 was positively correlated with the poor prognosis of male LUAD patients. On the basis of its association with the poor prognosis of the KRAS/TP53-mut group, we speculated that YTHDF1 plays an important role in the prognosis of KRAS/TP53-mut lung cancer. 

To investigate the mechanisms of YTHDF1 in KRAS- and TP53-mutant lung cancer, we firstly analyzed the expression of 237 proteins obtained from TCPA database. The results revealed that cyclin B1 was upregulated in terms of both protein level and translation efficiency in the KRAS/TP53-mut group. Cyclin B1 is a regulatory protein involved in mitosis and is necessary for the proper control of the G2/M transition phase of the cell cycle [20]. Research has demonstrated that degradation of CCNB1 is critical for cell-cycle progression and the proliferation of NSCLC cell lines [21]. In ovarian cancer cells, researchers found that the majority of YTHDF1-binding sites in CCNB1 are associated with the m^6^A sites [33]. In this study, we showed that the RNA level of YTHDF1 was positively associated with the protein level of cyclin B1. Additionally, the survival analysis demonstrated the consistent oncogenic role of CCNB1 in lung cancer. Our GSEA indicated that the pathway activity of the cell cycle in the KRAS/TP53-mut group was significantly upregulated, compared with the wildtype group. Pathways upregulated in the KRAS/TP53-mut group were associated with vital physiological functions, while pathways downregulated in the KRAS/TP53-mut group were enriched in metabolism, indicating the presence of metabolic disorders in the group. Hu et al. found that the SLC7A11/glutathione axis exerted metabolic synthetic lethality on oncogenic KRAS, indicating the presence of a metabolic disorder in KRAS-mutant lung cancer [44]. Therefore, all results demonstrated the critical regulatory effect of YTHDF1 on CCNB1 in the KRAS/TP53-mut group.

In this study, we observed the increased expression of YTHDF1 in KRAS and TP53 mutation cell lines. YTHDF1 downregulation contributed to inhibited tumor growth of lung cancer in vitro. In addition, we identified that YTHDF1 could regulate the proliferative ability of KRAS/TP53-mut lung cancer cells by promoting the translation of CCNB1 in vitro. These results revealed that YTHDF1 expression is associated with CCNB1 expression and may lead to the poor prognosis of KRAS/TP53-mut lung cancer patients. Similarly, YTHDF1 deficiency could inhibit NSCLC cell proliferation and xenograft tumor formation by regulating the translational efficiency of cell-cycle-related genes, such as CDK2, CDK4, and cyclin D1 [14]. Shi et al. found that YTHDF1 depletion restrains de novo lung adenocarcinoma (ADC) progression [14].

Recent advances in KRAS biology and structure have led to potential novel strategies for treating KRAS-mutant NSCLC, including selective small-molecule inhibitors, combination treatments, and immunotherapy [45]. An interesting report has shown that NSCLC patients treated with sotorasib, a KRAS^G12C^ inhibitor, achieved disease control leading to a median progress free survival (PFS) of 6.9 months. However, this current limited dataset suggests that neither KRAS p.G12C MAF nor PD-L1 expression level predicts response to sotorasib [46]. Meanwhile, tumor therapy targeting m^6^A modification has attracted researchers’ attention. For example, R-2HG, an inhibitor of demethylase FTO, combined with chemotherapy drugs, was found to exert synergistic anticancer effects against leukemia and glioma in vivo and in vitro [47,48]. In addition, YTHDF1 knockout in mice with colorectal cancer was found to lead to tumor shrinkage and prolonged survival [18]. Moreover, YTHDF1 knockout in classic dendritic cells was shown to increase the cross-presentation of tumor antigens and the cross-priming of CD8^+^ T cells [49]. YTHDF1′s relationship with the intestinal immune response might be mediated by the NF-κB signaling pathway [50]. These data suggest that YTHDF1 may be a potential therapeutic target in anticancer immunotherapy. However, the above evidence regarding therapy targeting m^6^A modification or YTHDF1 is at an early stage, and clinical trials are needed in future studies.

## 5. Conclusions

In summary, our study identified that enhanced expression of YTHDF1 is associated with the poor survival of LUAD patients with KRAS and TP53 co-mutation. YTHDF1 controls the translation of CCNB1 in an m^6^A-dependent manner and promotes the proliferation of KRAS/TP53-mut lung cancer. Thus, YTHDF1-targeted therapy may be a potential target for KRAS/TP53-mut lung cancer therapy.

## Data Availability

The genomic, transcriptomic, and clinical data of lung adenocarcinoma cancer were downloaded from the UCSC Xena browser (http://xena.ucsc.edu/, 14 December 2020). The protein data of lung adenocarcinoma cancer were downloaded from TCPA database (https://www.tcpaportal.org/, 17 December 2020). The GSE72094 was downloaded from the GEO database (https://www.ncbi.nlm.nih.gov/geo/query/acc.cgi?acc=GSE72094, 14 December 2020). Software and resources used for the analyses are described in the methods.

## Supplementary Materials

The following are available online at https://www.mdpi.com/article/10.3390/cells10071669/s1: Table S1. Lung adenocarcinoma cohort divided into the four mutation types; Table S2. Different types of mutations in LUAD samples in TCGA; Table S3. The protein expression level of 237 proteins in TCPA database; Table S4. The matched gene ID of the proteins from TCPA database; Table S5. The RNA expression level of the non-phosphorylated proteins; Table S6. The *p*-value of the non-phosphorylated proteins with a differential translation efficiency; Table S7. The *p*-value of the non-phosphorylated proteins with a differential protein level; Table S8. The upregulated pathways in the KRAS/TP53-mut group; Table S9. The upregulated pathways in the wildtype group; Table S10. Clinicopathologic characteristics of lung cancer patients.
